# Antiphospholipid antibodies in black south africans with hiv and acute coronary syndromes: prevalence and clinical correlates

**DOI:** 10.1186/1756-0500-4-379

**Published:** 2011-10-04

**Authors:** Anthony C Becker, Elena Libhaber, Karen Sliwa, Sham Singh, Simon Stewart, Mohammed Tikly, Mohammed R Essop

**Affiliations:** 1 Division of Cardiology, Chris Hani Baragwanath Hospital and University of the Witwatersrand, Johannesburg, South Africa; 2 Division of Rheumatology, Chris Hani Baragwanath Hospital and University of the Witwatersrand, Johannesburg, South Africa; 3 Preventative Cardiology, Baker IDI Heart and Diabetes Research Institute, Melbourne, Australia; 4 Lancet Laboratories, Johannesburg, South Africa

## Abstract

**Background:**

HIV infection is associated with a high prevalence of antiphospholipid antibodies (aPL) and increased thrombotic events but the aetiopathogenic link between the two is unclear.

**Findings:**

Prospective single centre study from Soweto, South Africa, comparing the prevalence of aPL in highly active anti-retroviral therapy (HAART) naïve HIV positive and negative patients presenting with Acute Coronary Syndromes (ACS). Between March 2004 and February 2008, 30 consecutive black South African HIV patients with ACS were compared to 30 black HIV negative patients with ACS. The HIV patients were younger (43 ± 7 vs. 54 ± 13, p = 0.004) and besides smoking (73% vs. 33%, p = 0.002) and lower HDL levels (0.8 ± 0.3 vs. 1.1 ± 0.4, p = 0.001) had fewer risk factors than the control group. HIV patients had a higher prevalence of anticardiolipin (aCL) IgG (47% vs. 10%, p = 0.003) and anti-prothrombin (aPT) IgG antibodies (87% vs. 21%, p < 0.001) but there was no difference in the prevalence of the antiphospholipid syndrome (44% vs. 24%, p = N/S) and aPL were not predictive of clinical or angiographic outcomes.

**Conclusions:**

Treatment naïve black South African HIV patients with ACS are younger with fewer traditional coronary risk factors than HIV negative patients but have a higher prevalence and different expression of aPL which is likely to be an epiphenomenon of the HIV infection rather than causally linked to thrombosis and the pathogenesis of ACS.

## Introduction

HIV infection is known to be associated with an increased prevalence of aPL but the link to the antiphospholipid syndrome (APS) with clinical thrombosis including myocardial infarction (MI) is tenuous [1]. Abuaf et al. [2] reported on the prevalence of aPL in HIV infection. Anticardiolipn antibodies (aCL) were reported to be present in 0-94%, anti-β_2-_glycoprotein-I (anti-β_2_-GPI) in 4-47%, anti-prothrombin (aPT) in 2-12% of patients with lupus anticoagulant (LA) found in 0-53.5%. Very few data exist on the prevalence of aPL in black patients especially aPT and IgA aPL isotypes in the setting of infections including HIV [3]. Loizou et al.[3] reported on the prevalence of aPL in 100 black South African HIV positive patients. There was a low prevalence of anti-β_2_-GPI (6%), all exclusively belonging to the IgA isotype, as well as aCL (7%), which were mainly positive for IgG. A prevalence of 43% (mainly IgG) aPT was found showing that the pattern of aPL in black South Africans differs from that found in caucasians [3]. Despite the unclear association between HIV, aPL and MI, there have been case reports in the non-HIV setting suggesting an association. A prospective case control analysis from the Honolulu Heart Program found that anticardiolipin antibodies, particularly the β_2_-Glycoprotein-dependent variety were strongly associated with the risk of MI [4]. In a study among survivors of MI, anticardiolipin antibodies were detected in 14% of patients compared with 3% of controls [5]. The antiphospholipid syndrome is found more commonly in populations with MI who have a low burden of conventional cardiovascular risk factors with little or no evidence of atherosclerotic disease and the condition needs to be considered in patients with normal appearing infarct related arteries [4]. We have shown that treatment-naïve HIV positive black South African patients presenting with acute coronary syndromes (ACS) are younger with fewer traditional risk factors compared to HIV negative patients and have less atherosclerotic burden but higher thrombotic burden on angiography [6]. In a subsequent study we showed that this group of HIV patients have evidence of thrombophilia as evidenced by lower protein C and higher factor VIII levels [7]. In addition, preliminary results showed a higher prevalence of aPL the significance of which is uncertain.

### Study Aims

It is within this context, that we investigated the role of aPL as a risk factor for ACS in treatment-naïve black South Africans with minimal traditional risk factors. We hypothesized that HIV patients with ACS, compared to HIV negative patients, would have a higher prevalence of aPL with a higher prevalence of the antiphospholipid syndrome (APS) and that aPL would be causally related to thrombosis and ACS.

## Methods

### Study design and patient enrollment

We conducted a prospective single centre study in the Department of Cardiology at Chris Hani Baragwanth Hospital, Soweto, South Africa. The protocol was approved by the ethics committee of the University of the Witwatersrand and adheres to the Declaration of Helsinki. All patients gave informed consent before study entry. Between March 2004 and February 2008, 30 consecutive black HIV patients presenting with ACS **(ACS+: HIV+ group) **were enrolled. For each HIV patient with ACS we selected the first presenting non-HIV black patient with ACS as a case-control comparator (**ACS+: HIV- group**). In addition a second control group consisting of 30 asymptomatic HIV patients matched for age, sex and ethnicity **(ACS-: HIV+ group) **were recruited from the HIV clinic. ACS was defined as either ST-elevation myocardial infarction, non ST-elevation myocardial infarction or unstable angina. Patients were categorized as having diabetes, hypertension or dyslipidemia when being treated chronically for these conditions or when diagnosed with the condition on admission. Patients were classified as having "other" coronary risk factors if any of the following conditions were present: i) Family history of premature CAD (men < 55 yrs, women < 65 yrs), ii) chronic kidney disease, iii) post menopausal state and iv) abdominal obesity (abdominal circumference > 102 cm in men and 88 cm in women). Demographic data was recorded for each patient and anthropometric measurements including weight, height, body mass index (BMI), waist to hip ratio and abdominal circumference (AC) were measured on admission according to guidelines set out in the Interheart study [8]. Infection with HIV was diagnosed with a standard enzyme linked immunosorbent assay and Western blot techniques after obtaining consent and offering pretest counseling. In the HIV group, Plasma HIV RNA level was determined by quantitative polymerase chain reaction. CD4 count was determined by flow cytometry and patients were staged according to the CDC staging system [9]. Patients with ACS were managed according to accepted guidelines set out by the European Heart Association [10,11] and followed up at the Chris Hani Baragwanath cardiac clinic.

### Laboratory methods

Blood was obtained by clean venipuncture with seated subjects and non-fasting venous blood was drawn into plastic tubes and allowed to clot at 37 degrees and then cetrifuged at 2500 × g for 8 minutes for sera preparation. The serum was immediately stored at -80 degrees until use. All sera were thawed only once in a water bath at 37 degrees for 15 minutes before testing. The analysis of aPL was performed at Lancet Laboratories, Johannesburg. Antiphospholipid antibodies including aCL, anti- β_2_-GP-1, and aPT (IgG, IgM, IgA) were measured at baseline in all patients and at least 12 weeks later in the ACS groups using commercial enzyme linked immunosorbent assay (ELISA) kits (Orgentec Diagnostika, Mainz, Germany). The results were expressed in units, according to the manufacturer's instructions. IgG, IgM and IgA aCL were expressed as U/mL. To establish normal values of aPL in our population we used the blood samples of 100 asymptomatic HIV negative black South African blood donors matched for age and sex to the study population. Results for each of the aPL measured were considered positive when the optical density obtained for each patient exceeded that of the mean value plus 2 standard deviations (SD) of the 100 sera from black South African normal healthy subjects. The normal values obtained were as follows: aCL IgG and IgA < 10 U/mL, IgM < 7 U/mL; anti- β_2_-GP-1 IgG, IgA, IgM < 8 U/mL; aPT IgG, IgA, IgM < 10 U/mL. The antiphospholipid syndrome (APS) was diagnosed in patients with ACS who had the presence of aPL (aCL IgG/IgM and/or anti- β_2_-GP-1 IgG/IgM) in titres greater than the mean plus 2SD from normal healthy subjects on two occasions at least 12 weeks apart [12].

### Stastistical analysis

Statistical analysis was performed using SAS 9.1 software (SAS, Cary, NC, USA). Normally distributed continuous data are presented as the mean (± standard deviation), and variables with non-Gaussian distribution as the median (min-max range). Categorical data are presented as frequencies and percentages. The initial analysis compared variables between the 3 groups using the one way anova test for continuous variables with normal distribution and the Kruskal Wallis test in case of non-normal distribution. For categorical variables the Chi square test was performed with a Fisher exact test when necessary. Significant differences between variables in the 3 groups was assumed at p < 0.05. Subgroup analysis with multiple pairwise comparisons was then performed applying the Bonferroni correction with a p value < 0.0166 considered significant. Univariate logistic regression was performed to determine predictors of the variables: aCL IgG, aPT IgG and APS: data presented as odds ratios (OR) with 95% confidence intervals (CI).

## Results

### Acute Coronary Syndrome (ACS+) group

The clinical profile and prevalence of aPL in the ACS+: HIV+ and ACS+: HIV-groups are listed in Table 1. HIV positive patients were younger with a similar sex distribution. Besides smoking, (33% vs. 73%, p = 0.004), coronary risk factors were higher in the ACS+: HIV- group with more hypertension (p = 0.0001), LDL hyperlipidaemia (p = 0.003), diabetes mellitus (p = 0.03) and "other coronary risk factors" (p = 0.0001). The ACS+: HIV+ group had lower HDL levels (p = 0.0006) and a lower mean BMI (p = 0.008). The ACS+: HIV+ group had higher frequencies of aCL IgG (47% vs. 10%, p = 0.003) and aPT IgG (87% vs. 21%, p < 0.001) compared to the ACS+: HIV- group. There were no differences between groups with respect to the other aPL frequencies. The ACS group (60 patients) was then analysed to determine the prevalence of APS. In the ACS+: HIV+ group, 18/30(60%) patients had aPL results from two separate occasions, 12 weeks apart as per diagnostic guidelines [12]. 12/30(40%) did not have repeat testing owing to 9/30(30%) deaths and 3/30(10%) patients lost to follow up. In the ACS+: HIV- group, 25/29(86%) patients had aPL results from two separate occasions, 12 weeks apart with 4/29(14%) patients dying prior to repeat testing. The diagnostic criteria for APS were met in 8/18(44%) HIV patients vs. 6/25(24%) HIV negative patients, p = 0.28. In the ACS+: HIV+ group, 7/8(88%) had aCL IgG antibodies, 1/8(13%) aCL IgM, 6/8(75%) anti- β_2_-GP-1 IgG and 1/8(13%) anti- β_2_-GP-1IgM. 5/8(63%) patients had the presence of both aCL IgG and anti- β_2_-GP-1IgG antibodies. In the ACS+: HIV- group, 3/6(50%) had aCL IgG antibodies, 1/6(17%) aCL IgM and 4/6(67%) anti- β_2_-GP-1IgG. 2/6(33%) had the presence of both aCL IgG and anti- β_2_-GP-1IgG antibodies.

**Table 1:** ACS Group: Clinical profile and aPL prevalence

	ACS+: HIV+	ACS+: HIV-	p value
**Demographic Profile:**			

Black African n(%)	30(100)	30(100)	1.0

Mean Age (years)	43 ± 7	54 ± 13	< 0.001

Men (%)	20(67)	18(60)	N/S

**Coronary risk factors n(%):**			

Smoking	22(73)	10(33)	0.004

Diabetes Mellitus	1(3)	7(23)	0.05

Hypertension	7(23)	23(77)	< 0.001

Total cholesterol (mmol/l)	3.6 ± 1.0	4.6 ± 1.4	0.003

LDL cholesterol (mmol/l)	2.2 ± 0.9	3.0 ± 1.2	0.003

HDL cholesterol (mmol/l)	0.8 ± 0.3	1.1 ± 0.4	< 0.001

Triglycerides (mmol/l)	1.4 ± 0.8	1.1+/-0.4	N/S

BMI (kg/m^2^)	25 ± 5	28 ± 5	0.008

Other coronary risk factors	2(7)	16(53)	< 0.001

**Baseline aPL frequencies(%):**			

Blood samples n(%)	30(100)	29(97)*	N/S

Anticardiolipin (IgG)	14(47)	3(10)	0.003

Anticardiolipin (IgM)	3(10)	1(3)	N/S

Anticardiolipin (IgA)	0(0)	3(10)	N/S

Beta-2 Glycoprotein (IgG)	11(37)	6(21)	N/S

Beta-2 Glycoprotein (IgM)	3(10)	0(0)	N/S

Beta-2 Glycoprotein (IgA)	7(23)	12(41)	N/S

Anti-Prothrombin (IgG)	26(87)	6(21)	< 0.001

Anti-Prothrombin (IgM)	2(7)	0(0)	N/S

Anti-Prothrombin (IgA)	2(7)	1(3)	N/S

**Antiphospholipid syndrome (%):**	8/18(44)	6/25(24)	N/S

**Legend: **Data are presented as the mean ± standard deviation or proportions

**Key: **BMI = body mass index

aPL = antiphospholipid antibody

* 1 patient not analysed due to an inadequate blood sample

### HIV (HIV+) group

The clinical profile and prevalence of aPL in the ACS+: HIV+ and ACS-: HIV+ groups are listed in Table 2.

**Table 2:** HIV group: Clinical profile and aPL prevalence

	ACS+: HIV+	ACS-: HIV+	p value
**Demographic Profile:**			

Black African n(%)	30(100)	30(100)	1.0

Mean Age (years)	43 ± 7	41 ± 8	N/S

Men (%)	20(67)	19(63)	N/S

**HIV related factors:**			

CD4(cells/mm^3^) median(range)	230 (30 -1356)	125 (6-1041)	0.013

Viral load (RNA copies/ml) median(range)	29000 (25 - 7 × 10^5^)	54000(25-11 × 105)	N/S

AIDS defining criteria n(%)	11(37)	21(70)	0.01

Current oppurtunistic infection	0	1(3)	N/S

HIV related malignancies	0	2(7)	N/S

**Coronary risk factors n(%):**			

Smoking	22(73)	11(37)	0.003

Diabetes Mellitus	1(3)	0(0)	N/S

Hypertension	7(23)	2(7)	N/S

Total cholesterol (mmol/l)	3.6 ± 1.0	3.7+/-0.8	N/S

LDL cholesterol (mmol/l)	2.2 ± 0.9	2.0 ± 0.5	N/S

HDL cholesterol (mmol/l)	0.8 ± 0.3	1.0 ± 0.5	0.011

Triglycerides (mmol/l)	1.4 ± 0.8	1.4+/-0.8	N/S

BMI (kg/m^2^)	25 ± 5	21 ± 4	0.003

Other coronary risk factors	2(7)	0(0)	N/S

**Baseline aPL frequencies (%):**			

Blood samples n(%)	30(100)	30(100)	1.0

Anticardiolipin (IgG)	14/30(47)	17/30(57)	N/S

Anticardiolipin (IgM)	3/30(10)	4/30(13)	N/S

Anticardiolipin (IgA)	0/30(0)	0/30(0)	N/S

Beta-2 Glycoprotein (IgG)	11/30(37)	10/30(33)	N/S

Beta-2 Glycoprotein (IgM)	3/30(10)	2/30(7)	N/S

Beta-2 Glycoprotein (IgA)	7/30(23)	8/30(27)	N/S

Anti-Prothrombin (IgG)	26/30(87)	29/30(97)	N/S

Anti-Prothrombin (IgM)	2/30(7)	0/30(0)	N/S

Anti-Prothrombin (IgA)	2/30(7)	3/30(10)	N/S

**Legend: **Data are presented as the mean ± standard deviation or proportions

**Key: **BMI = body mass index

aPL = antiphospholipid antibody

The ACS+: HIV+ and ACS-: HIV+ groups were well matched with respect to age, sex and viral load. The ACS+: HIV+ group were less immunocompromised as evidenced by higher CD4 counts (p = 0.013) and less patients with AIDS defining criteria (p = 0.01) which were all based on a CD4 count < 200 cells/ml^3^. There were no opportunistic infections or HIV related malignancies in the ACS+: HIV+ group and 18/30(60%) patients had early disease being classified as either stage A1 or A2 [9]. In the ACS-: HIV+ group, 21/30(70%) patients had AIDS, 18/30(60%) due to a CD4 count < 200 cells/ml^3 ^and 3/30(10%) patients with either oppurtunistic infections or AIDS related malignancies: 1/30(3%) patient having active pulmonary Tuberculosis, 1/30(30%) with cutaneous Kaposi's Sarcoma and 1/30(3%) with a Non-Hodgkins Lymphoma. 6/30(20%) had early disease (stage A1 or A2). In terms of coronary risk factors, there were more smokers in the ACS+: HIV+ group (p = 0.0026) and the mean HDL levels were lower (p = 0.011) compared to the ACS-: HIV+ group. There were no significant differences between the groups with respect to aPL frequencies but analysis of the actual aPL titres revealed higher levels of β_2_-GP-1 IgM [3.0(1.6-10.2) vs. 2.1(1.3-15.3), p = 0.007] in the ACS-: HIV+ group compared to the ACS+: HIV+ as well as aPT IgG [20.2(9.3-165.1) vs. 14.4(7.5-40.2), p = 0.0008], aPT IgM [4.8(1.3-9.4) vs. 3.1(1.4-30.9), p = 0.012] and aPT IgA [5.8(3.3-17.3) vs. 4.6(3.0-18.2), p = 0.015]. On univariate logistic regression, HIV infection was predictive of the presence of aCL IgG (OR 2.94, CI 1.60-5.40) and aPT IgG antibodies (OR 16.07, CI 5.38-49.94) but did not predict APS (OR 2.53, CI 0.69-9.36). The presence of aPL's (aCL IgG, aPT IgG) and APS did not predict any of the clinical or angiographic outcomes in the study including markers of atherosclerotic and thrombotic burden.

## Discussion

HIV infection is known to be associated with increased frequencies of aPL [13] and the antiphospholipid syndrome is found more commonly in populations with ACS who have a low burden of conventional cardiovascular risk factors with little or no evidence of atherosclerotic disease [4] such as our HIV cohort. No data currently exists, however, on aPL frequencies and clinical correlates in HAART-naïve HIV patients. We took the almost unique opportunity to study a HAART-naïve population thus negating the effects of HAART on thrombotic risk. Firstly, when comparing the ACS+: HIV+ group and the ACS+: HIV- group, consistent with previously published data, the HIV group were younger in age, predominantly male, with a higher percentage of cigarette smokers and lower HDL levels [14]. Besides smoking, there were less traditional risk factors for ACS in the ACS+: HIV+ group. With respect to aPL frequencies in the ACS group, significant differences were found with HIV patients having higher frequencies of aCL IgG and aPT IgG antibodies. The incidence of APS was, however, similar in both groups. What is the significance of these findings? The exact role of aPL in the aetiopathogenesis of thrombosis in HIV infected patients is controversial. Despite the findings of high aPL frequencies [2] and case reports of APS in HIV patients, antiphospholipid antibodies often present in infectious diseases are not usually associated with thrombotic complications [3]. In order to address the question of significance of the aPL findings in our HIV patients with ACS, we looked at a similar HIV group matched for age, sex and degree of immunosuppression (ACS-: HIV+ group). The ACS+: HIV+ group, had a different pattern of aPL expression compared to recent studies investigating a predominantly caucasian population which reported aCL to be positive in 36-88%, anti- β_2_-GP-1 in 4-27%, and aPT in 2-12% of patients [13,15-17]. In keeping with the study by Loizou et al [3] we found a high prevalence of aPT IgG antibodies in our black HIV cohort with 87% in the ACS+: HIV+ group and 97% in the ACS-: HIV+ group. The rates of aCL and anti- β_2_-GP-1 antibodies seen in our black HIV population were similar to those described in the recent studies on Caucasian patients [13,15-17]. Comparing the aPL frequencies within the HIV group, there were no statistically significant differences between the ACS+: HIV+ and ACS-: HIV+ groups but analysis of the actual aPL titres revealed higher levels of β_2_-GP-1 IgM as well as aPT (IgG, IgM, IgA) in the ACS-: HIV+ group possibly due to a greater degree of immunosuppresion and immune dysregulation [3], suggesting that the higher frequencies of aPL seen in HIV patients are an epiphenomenon of HIV infection rather than causally linked to thrombotic events. The aPL seen in HIV patients could be induced by disturbances in regulation of cellular and humoral immunity, as a secondary consequence of HIV infection. Alternatively, their induction might result from the exposure of cell wall phospholipids as a consequence of damaged body cells resulting from the HIV-related inflammatory milieu [3]. Several limitations of the study require comment. Although the study constitutes one of the largest prospective analyses on treatment-naïve patients with ACS, the sample size in each group was relatively small resulting in a lack of power to detect small differences between the groups which may have been significant. In addition, not all patients had repeat testing at 12 weeks which would have influenced the reported incidence of APS in the patients who had thrombotic events. Wherever possible, however, we have adhered to the recently published STROBE guidelines in our reporting of study data [18].

## Conclusions

Treatment-naïve HIV patients presenting with ACS have different risk factors and clinical features compared to the HIV negative population as well as a higher prevalence and different pattern of aPL expression. It is unlikely that aPL in this group of patients are causally linked to ACS and are more likely an epiphenomenon of HIV infection. It is possible that the pathogenesis of thrombosis in these patients is multifactorial with the interaction of conventional risk factors and other HIV-specific coagulation abnormalities which warrants further study. To this extent smoking is an important modifiable risk factor and should be an important target for cardiovascular risk reduction. Routine screening for antiphospholipid antibodies in HIV patients presenting with ACS and minimal risk factors cannot be justified based on these findings.

## Competing interests

The authors declare that they have no competing interests.

## Authors' contributions

AB conceived and designed the research, acquired, analysed and interpreted the data and drafted the manuscript. EL analysed and interpreted the data, performed statistical analysis and helped in drafting the manuscript. KS helped in the research design, data interpretation and analysis, drafting the manuscript and handled funding and supervision. Sham Singh helped in data acquisition, interpretation as well as drafting the final manuscript. Simon Stewart helped in research design, analysis and interpretation of the data, statistical analysis and drafting of the manuscript. MT participated in the conception and design of the research, analysis and interpretation of data as well as final manuscript preparation. ME participated in the conception and design of the research, data analysis and interpretation, research supervision and drafting of the final manuscript. All authors read and approved the final manuscript.
